# Rapid elimination of CO through the lungs: coming full circle 100 years on

**DOI:** 10.1113/expphysiol.2011.059428

**Published:** 2011-10-03

**Authors:** Joseph A Fisher, Steve Iscoe, Ludwik Fedorko, James Duffin

**Affiliations:** 1 Department of Anesthesiology, University Health Network, University of TorontoToronto, Canada; 2 Department of Biomedical and Molecular Sciences, Queen's UniversityKingston, Canada; 3 Hyperbaric Medicine Unit, University Health NetworkToronto, Canada

## Abstract

At the start of the 20th century, CO poisoning was treated by administering a combination of CO_2_ and O_2_ (carbogen) to stimulate ventilation. This treatment was reported to be highly effective, even reversing the deep coma of severe CO poisoning before patients arrived at the hospital. The efficacy of carbogen in treating CO poisoning was initially attributed to the absorption of CO_2_; however, it was eventually realized that the increase in pulmonary ventilation was the predominant factor accelerating clearance of CO from the blood. The inhaled CO_2_ in the carbogen stimulated ventilation but prevented hypocapnia and the resulting reductions in cerebral blood flow. By then, however, carbogen treatment for CO poisoning had been abandoned in favour of hyperbaric O_2_. Now, a half-century later, there is accumulating evidence that hyperbaric O_2_ is not efficacious, most probably because of delays in initiating treatment. We now also know that increases in pulmonary ventilation with O_2_-enriched gas can clear CO from the blood as fast, or very nearly as fast, as hyperbaric O_2_. Compared with hyperbaric O_2_, the technology for accelerating pulmonary clearance of CO with hyperoxic gas is not only portable and inexpensive, but also may be far more effective because treatment can be initiated sooner. In addition, the technology can be distributed more widely, especially in developing countries where the prevalence of CO poisoning is highest. Finally, early pulmonary CO clearance does not delay or preclude any other treatment, including subsequent treatment with hyperbaric O_2_.

## Background

At the turn of the 20th century, CO poisoning was treated by administering high concentrations of O_2_ to increase the O_2_ carried in the blood and, if necessary, ventilation was stimulated by adding CO_2_. It was initially and mistakenly thought that patients asphyxiated to unconsciousness by CO had a total body deficit of CO_2_ that was replenished by the inhaled CO_2_ (Henderson *et al.* 1921). Furthermore, animal tests had shown that the addition of CO_2_ to O_2_ markedly increased the dissociation of carboxyhaemoglobin (COHb) and accelerated clearance of CO compared with using O_2_ alone (Henderson & Haggard, 1920). Carbon dioxide was administered in concentrations of 5–10% in O_2_, known as ‘carbogen’. From the very beginning, treatment of CO-poisoned patients with carbogen at the site of rescue led to reports of dramatic reversals of coma and other neurological symptoms (Henderson & Haggard, 1922). In short order, the administration of carbogen became the standard of care for CO poisoning, and remained so for almost a half-century. Indeed, carbogen remains a stock item in many hospitals to this day.

### Hyperbaric oxygen

By the 1960s, the rationale for using carbogen for CO poisoning was increasingly questioned (Donald & Paton, 1955). The notion that CO poisoning was accompanied by a deficit of CO_2_ was rejected (Donald & Paton, 1955). Ventilatory stimulation by CO_2_ was no longer required, because hypoventilation accompanying coma could be managed by endotracheal intubation and mechanical ventilation. It became feasible to increase CO dissociation from haemoglobin (Hb) by exploiting the mass action effect of O_2_ on the equilibrium (Haldane, 1895) COHb + O_2_ ⇆ O_2_Hb + CO by administering the O_2_ at hyperbaric pressures (Pace *et al.* 1950). Hyperbaric O_2_ replaced carbogen as the preferred treatment (Smith, 1962) because it was thought (mistakenly, as subsequently demonstrated; Fisher *et al.* 1999) to result in faster CO elimination (Norman & Ledingham, 1967) and, on theoretical grounds, to be effective at reversing the assumed toxic effects of CO in such extravascular tissues as the brain (Brown & Piantadosi, 1990; Stoller, 2007).

### Time to treatment over type of treatment

The point cannot be too strongly emphasized that for treatment to be effective it must be applied at the earliest possible moment after the victim is discovered, and must remove the carbon monoxide from his blood as soon as possible. (Henderson & Haggard, 1922)

Although the physics and chemistry underpinning the effectiveness of hyperbaric O_2_ in clearing CO from the blood are unassailable, and some beneficial effects can be demonstrated in animals (Brown & Piantadosi, 1990, 1992; Piantadosi *et al.* 1997), in practice it has been difficult to demonstrate its clinical efficacy. The poor response of most victims of CO poisoning to hyperbaric O_2_ has been confirmed repeatedly by expert panels in Australia, Canada and the USA (Buckley *et al.* 2005; Juurlink *et al.* 2005; McMaster University Division of Emergency Medicine, 2006; Wolf *et al.* 2008), as well as large controlled trials in Australia (Scheinkestel *et al.* 1999) and France (Annane *et al.* 2010). The primary lesson to be learned from the discrepancies between animal and clinical studies is that for patients poisoned by CO, the time to treatment, rather than the method of treatment, is of major importance (Gorman *et al.* 1992; Scheinkestel *et al.* 1999). Even from the very beginning of hyperbaric O_2_ treatment of CO poisoning in Glasgow, it was clear that delays between poisoning and treatment markedly reduced its effectiveness (Smith, 1962). Times to treatment as short as 3–6 h, which are all that can be expected for hyperbaric O_2_ given the logistics of patient transport and chamber preparation, continue to show no benefit compared with normobaric O_2_ (Scheinkestel *et al.* 1999; Annane *et al.* 2010).

### Effect of time to treatment on pathology of CO poisoning

It has been long understood that ‘asphyxia is not immediately terminated when the victim is removed from the gassing chamber…although his body may be surrounded and his lungs filled with fresh air, his brain continues to be asphyxiated’ (Henderson & Haggard, 1922). Eventually, there is a redistribution of CO from blood to extravascular tissues (Coburn, 1970), drawn there by the high affinity of some cellular molecules for CO [e.g. myoglobin in heart muscle (Coburn, 1970; Dolan, 1985) and cytochromes in the brain (Cronje *et al.* 2004)], even at low [COHb], and particularly with hypoxaemia (Dolan, 1985).

**Figure 1:**
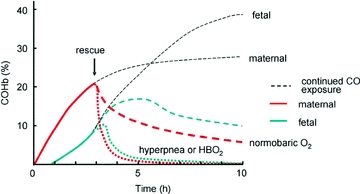
Schematic diagram illustrating the kinetics of [COHb] in mother (red) and fetus (teal) after 3 h exposure to CO and then rescue Black dotted lines represent [COHb] at equilibrium; coloured dashed lines represent [COHb] with normobaric O_2_ treatment; coloured dotted lines represent [COHb] with accelerated CO clearance. (Figure from Rucker & Fisher, 2006, with permission. Labels added to original figure by the authors.)

One instructive model of CO distribution kinetics to an extravascular compartment is CO in the fetus, as studied by Longo and colleagues (Hill *et al.* 1977; Longo & Hill, 1977) in pregnant sheep. Fetal Hb has a higher affinity for both O_2_ and CO than maternal Hb. After an initial maternal exposure to CO, there is a delay in the transfer of CO to the fetus of about 1 h (Longo & Hill, 1977), which is characteristic of many tissues (Cronje *et al.* 2004). This delay is due to the low partial pressure of CO (*P*_CO_) in the plasma, because it is tightly bound to Hb (Bruce *et al.* 2008). Eventually, at higher [COHb], *P*_CO_ rises and CO begins to diffuse into the tissues. At equilibrium, fetal [COHb] will exceed maternal [COHb] (dotted lines in Fig. 1). If rescue occurs prior to equilibration of CO, maternal [COHb] will follow the time course illustrated in Fig. 1. If normobaric O_2_ is administered, the maternal half-time of CO elimination will be ∼80 min (Dolan, 1985). However, because of the greater affinity of fetal Hb for CO, fetal [COHb] will continue to rise and so exceed that of the mother, even as her [COHb] is falling. If CO clearance from the mother is accelerated, the *P*_CO_ gradient between the fetus and mother increases (Longo & Hill, 1977), thereby also increasing the rate of elimination from the fetus. A computer simulation of CO kinetics between mother and fetus using the model proposed by Hill & Longo (1977) is available as a supplemental file entitled CO Model.zip.

These principles of CO kinetics have long been acknowledged (Henderson & Haggard, 1922; Smith, 1962; Scheinkestel *et al.* 1999); yet somehow, by consensus, a treatment that was highly effective because it could be administered with the least delay (carbogen) was abandoned for another (hyperbaric O_2_) despite its associated delay in treatment. The (presumed) greater rate of CO elimination and the potential of reversing CO-related pathology (Sharp *et al.* 1962) with hyperbaric O_2_ was considered an acceptable trade-off for the difficult logistics, increased expense and added delay in treatment. Despite little evidence of its value, hyperbaric O_2_ has remained the mainstay of treatment for the last half a century.

### Is normobaric oxygen a standard of care?

**Figure 2:**
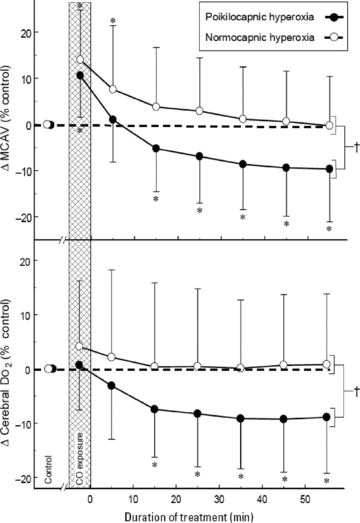
Effect of poikilocapnic and isocapnic normobaric O_2_ treatment on cerebral O_2_ delivery (DO_2_) in CO-exposed human subjects Fourteen human subjects were exposed on two separate occasions to CO until their [COHb] reached 10–12%. They were administered 100% O_2_ with, and without, maintaining isocapnia. Blood O_2_ delivery was calculated from arterial PO_2_, arterial *P*_CO_2__, haemoglobin saturation (11–[COHb]), plasma O_2_ content, and changes in middle cerebral artery flow velocity (MCAV) as measured by transcranial Doppler (as a surrogate of change in cerebral blood flow). Poikilocapnic hyperoxia resulted in a significantly lower DO_2_. (From Rucker *et al.* 2002, with permission from the publisher.)

Even normobaric O_2_ treatment of CO poisoning is problematic. The effect of *P*_CO_2__ on the half-time of [COHb] reduction in patients treated in hospital (as opposed to laboratory volunteers) is highly unreliable (*r*^2^= 0.19), ranging from 26 to 148 min (Weaver *et al.* 2000). Furthermore, normobaric O_2_ treatment may even contribute to the morbidity of CO poisoning. Apart from the potential for free radical generation by hyperoxia (Thom, 1990), there is also the underappreciated effect of hyperoxia as a ventilatory stimulant. Hyperoxia-induced hyperventilation results in some degree of hypocapnia (Becker *et al.* 1996), which is associated with a reduction of blood flow in such CO_2_-responsive vascular beds as the coronary (Case *et al.* 1975) and cerebral circulations. The reduction in cerebral (Kety & Schmidt, 1948) blood flow with hypocapnia occurs even in the presence of increased levels of CO in the blood (Rucker *et al.* 2002). In normoxic individuals, as well as those with high [COHb] (Henderson & Haggard, 1922), normobaric O_2_ produces only a very small increase in blood O_2_ content that is carried in the plasma, where it is poorly soluble. If this small increase in blood O_2_ content is accompanied by even a small reduction in tissue blood flow, the result can be a net reduction in organ O_2_ delivery (Case *et al.* 1975; Rucker *et al.* 2002). Figure 2 illustrates that the administration of normobaric O_2_, an undisputed treatment for CO poisoning since the time of Haldane (Haldane, 1895), may even exacerbate the brain ischaemia resulting from CO poisoning.

## Back to the future

If there are problems with carbogen, hyperbaric and normobaric O_2_, where do we go from here?

### Increased alveolar ventilation can be as effective as hyperbaric O_2_

**Figure 3:**
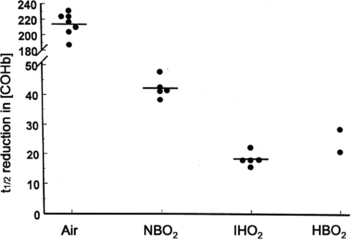
Elimination half-times for [COHb] Five anaesthetized, intubated, spontaneously breathing dogs were exposed to CO until [COHb] reached ∼70%. They were then administered, sequentially, room air (Air), normobaric O_2_ (NBO_2_) and then vigorously mechanically ventilated with O_2_ while maintaining normocapnia (IHO_2_). Blood was drawn every 5 min and analysed for [COHb]. Plots of log [COHb]*versus* time were used to calculate the half-times of reduction in [COHb]. Values are compared with dogs prepared in a similar manner and treated with normocapnic ventilation with O_2_ at 3 atm (304 kPa). Isocapnic hyperpnoea resulted in a similar rate of [COHb] reduction to hyperbaric O_2_ (HBO_2_). Reprinted with permission of the American Thoracic Society. Copyright © American Thoracic Society. Hyperbaric data from the original study reported in the text was added to the figure by the authors.

About a decade ago, the trade-offs between rate of CO elimination and time to treatment were re-examined. The initial studies compared the half-times of reduction of [COHb] induced by increases in alveolar ventilation with those resulting from hyperbaric O_2_. Previous studies (Henderson & Haggard, 1920) had concentrated on the relative efficacies of various mixtures of CO_2_ in O_2_ for reducing [COHb] in spontaneously breathing animals (Walton *et al.* 1925) and humans (Henderson & Haggard, 1922). In the early 1960s, it became apparent that the elimination of rebreathing during assisted ventilation (Douglas *et al.* 1961) and the magnitude of the minute ventilation (Killick & Marchant, 1959), i.e. the net alveolar ventilation, rather than the concentration of CO_2_ in the carbogen, was the main factor determining the half-time of elimination. Indeed, with controlled ventilation Fisher *et al.* (1999) demonstrated, in dogs, that isocapnic increases in alveolar ventilation result in the same half-times of CO elimination as those for hyperbaric O_2_ (Fig. 3).

### Favourable CO kinetics with increased alveolar ventilation

**Figure 4:**
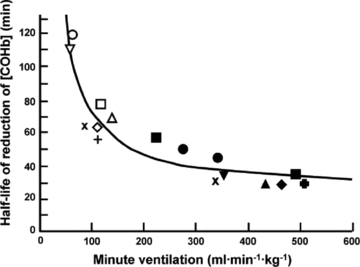
Half-time of COHb reduction *versus* minute ventilation in humans Seven men were exposed to CO until [COHb] reached 10–12% on two separate occasions. On one occasion, subjects breathed 100% O_2_ (‘resting ventilation’). On the other occasion, subjects were administered 100% O_2_ and asked to increase their minute ventilation; on that occasion, isocapnia was maintained. Venous blood was drawn every 5 min and analysed for [COHb]. Open symbols represent values during resting ventilation (normobaric O_2_); filled symbols during normocapnic hyperpnoea. Half-times of elimination were calculated from plots of log [COHb]*versus* time. Most of the increase in [COHb] reduction was reached at a relatively modest 200 ml min^−1^ kg^−1^, or 14 1 min^−1^ for a 70 kg person. (From Takeuchi *et al.* 2000; reprinted with permission of the American Thoracic Society. Copyright © American Thoracic Society.)

Takeuchi *et al.* (2000) then investigated CO elimination half-times in spontaneously breathing human volunteers exposed to CO. Subjects breathed O_2_ using a circuit that maintained normocapnia. Several findings from this study are of interest. First, the ventilatory response to normobaric O_2_ (open symbols in Fig. 4) varied between subjects. Second, the relationship between elimination half-times and minute ventilation is a rectangular hyperbola. This shape means that initial graded increases in minute ventilation above resting values result in the greatest reductions in half-times. For example, a 70 kg patient ventilating at about 15–20 l min^−1^ (levels easily tolerated by patients without severe lung disease) can reduce the half-time to a value similar to that reported for hyperbaric O_2_ (Takeuchi *et al.* 2000). Finally, the relationship between minute ventilation and elimination half-time is scalable to body size and sex (Tesler, 2000).

### Back to carbogen?

Is it therefore appropriate to resurrect carbogen as a readily deployable means to increase alveolar ventilation without reducing arterial *P*_CO_2__? Unfortunately, it is not. As early as 1955, an official report to the Medical Research Council (UK) (Donald & Paton, 1955) warned about the risk of exacerbating acidosis by administering carbogen to patients who are already retaining CO_2_ due to ventilatory depression from severe CO poisoning or previously ingested drugs. As for those patients with an intact ventilatory response to CO_2_, administration of CO_2_ up to a concentration of 4% increases the minute ventilation only by a factor of two (Soley *et al.* 1941), thereby limiting its effectiveness in CO elimination. Moreover, large individual variations in ventilatory responses to inhaled CO_2_ (Solely *et al.* 1941; Prisman *et al.* 2007) mean that one cannot guarantee an increased rate of CO elimination, or even that hypocapnia will be prevented (Baddeley *et al.* 2000; Prisman *et al.* 2007). Above an inspired CO_2_ concentration of 4%, minute ventilation markedly increases, but so does respiratory distress (Baddeley *et al.* 2000); these investigators found that 30% of patients and healthy subjects were unable to tolerate 5% CO_2_. It is therefore unlikely that a single premixed carbogen dose will fit all.

### Hyperpnoea without carbogen

It follows from the preceding discussion that exploiting an increase in alveolar ventilation to clear the blood of CO will require a different approach. The method used must maintain normocapnia in order to allow patients to sustain increased ventilation comfortably for two to three half-times of CO elimination, thereby achieving more complete elimination of CO. Rather than administering a fixed concentration of CO_2_ in an attempt to maintain normocapnia with hyperpnoea, one can administer CO_2_ in direct proportion to increases in minute ventilation above basal levels (Sommer *et al.* 1998). Ideally, the apparatus that would be used to maintain normocapnia would be safe, easy to use, portable and, if at all possible, inexpensive.

### Increasing alveolar ventilation while maintaining normocapnia

**Figure 5:**
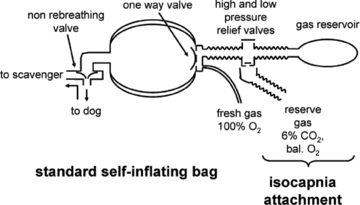
A self-inflating bag circuit suitable for spontaneous and controlled ventilation Reserve gas enters circuit through the inspiratory relief valve of the self inflating bag (modified from Fig. 1 of Sasano *et al.* 2001; figure reproduced with permission of the publisher.)

**Figure 6:**
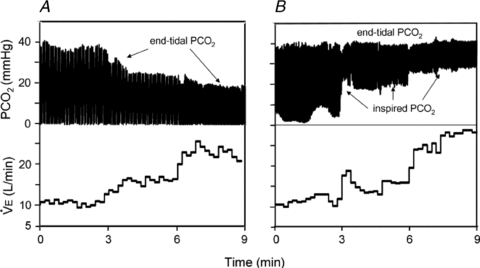
Data from a human subject to illustrate the effect of voluntary hyperventilation, without (*A*) and with maintenance of normocapnia (*B*) with the breathing circuit depicted in **Fig. 6**, on end-tidal *P*_CO_2__ Lower panels show minute ventilation (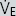) and upper panels show continuous capnograph traces. Peaks are end-tidal *P*_CO_2__ and troughs represent inspired *P*_CO_2__. Note proportional increases in inspired *P*_CO_2__ as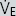 increases; end-tidal *P*_CO_2__ remains unchanged, regardless of 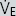.

Historically, the advances in treatment of CO poisoning were also linked to the fabrication of devices required to implement them. Henderson and Haggard in New York devised their H-H Infusor to administer carbogen (Henderson & Haggard, 1922). Smith and Sharp (1960) built the first fixed and then portable hyperbaric chambers (Norman *et al.* 1970) in the Aberdeen Royal Infirmary, in Scotland. Recently, researchers in our laboratory (Sommer *et al.* 1998) described a method that passively maintains normocapnia regardless of minute ventilation and pattern of breathing. In that circuit, a constant O_2_ flow is provided to a standard self-inflating bag, and the inspiratory relief valve of the self-inflating bag is attached to a demand regulator supplying 6% CO_2_ in O_2_ (Fig. 5). Any increase in minute ventilation above the O_2_ flow is therefore supplied by the demand regulator (6% CO_2_ in O_2_). The O_2_ flow is adjusted to match the patient's metabolic CO_2_ production and controls the alveolar ventilation for CO_2_. Arterial *P*_CO_2__ is therefore unchanged by any increase in ventilation, because any ventilation exceeding the O_2_ flow is composed of 6% CO_2_ in O_2_, a mixture that does not contribute to a CO_2_ diffusion gradient between capillary blood and the alveoli (Sommer *et al.* 1998; Somogyi *et al.* 2005; Fig. 6). However, it is the combined flow of O_2_ and 6% CO_2_ in O_2_ that serves to wash out CO from the lungs, thereby clearing it from the blood.

The system is designed to be used in the field, but it cannot be readily improvised and requires deliberate preparation. It requires a customized breathing circuit or modification of available self-inflating bags, compressed CO_2_-containing gas with specific pressure regulator and flow controller. Such tanks require care in storage or use in extreme cold because CO_2_ liquefies readily when cold. Use of the system requires some clinical expertise or monitoring of end-tidal gas in order to set the fresh gas flow (O_2_ or air) appropriately to attain an appropriate end-tidal *P*_CO_2__. However, due to the benign nature of acute hypercapnia in adults (Potkin & Swenson, 1992; Ayas *et al.* 1998), as well as in children (Goldstein *et al.* 1990), when oxygenation is maintained, the fresh gas flow need not be exact and can be safely titrated to comfort or ventilatory response, or can be set according to guidelines based on approximate body weight.

### Isocapnic hyperpnoea in practice

We suggest that the availability of a portable device to increase CO clearance would be a useful adjunct to current treatment of CO poisoning. It can be brought to the field to begin treatment immediately at the time of rescue and continue treatment during transportation to hospital. The same device can be applied to patients breathing spontaneously, as well as those requiring ventilatory assistance. Prior CO clearance at the site of rescue would make emergency air transport safer, should it be required. As normocapnia is maintained and there are no foreseeable risks, this treatment can be administered on the suspicion of CO poisoning. It would therefore provide the earliest possible treatment if CO poisoning is later confirmed, and nothing is lost if it is not. Carbon monoxide poisoning often occurs in clusters, and this treatment approach can be inexpensively and safely applied to all victims. Finally, early pulmonary CO clearance does not delay or preclude any other treatment, including subsequent treatment with hyperbaric O_2_, if deemed necessary (Piantadosi, 2002; Weaver *et al.* 2002).

It is also noteworthy that isocapnic increases in alveolar ventilation with 21% O_2_ would be as effective in eliminating CO as normobaric hyperoxia (Henderson & Haggard, 1920), yet avoid risk of the additional oxidative stress from hyperoxia. Furthermore, both hyperoxic and normoxic isocapnic hyperpnoea would also accelerate the clearance of any volatile hydrocarbons, including ethanol (Henderson, 1924; Hunter & Mudd, 1924), methanol, ingested poisons (Lemburg *et al.* 1979) and anaesthetic agents (Sasano *et al.* 2001; Vesely *et al.* 2003; Katznelson *et al.* 2008, 2010).

## Summary

We believe we have now come full circle in the treatment of CO poisoning. At the beginning of the 20th century, carbogen proved to be an effective means of treating CO poisoning. Only relatively recently was it realized that it was not the CO_2_ in carbogen but the increase in alveolar ventilation induced by the CO_2_ that accelerated the clearance of CO. By then, however, rapid advances in the technology of positive-pressure ventilation and hyperbaric chambers overshadowed the old-fashioned approach using carbogen. Despite the initial enthusiasm for hyperbaric O_2_ as the treatment for CO poisoning, the fact remains that hyperbaric O_2_ facilities are expensive and their distribution around the world is poorly matched to the incidence and prevalence of CO poisoning. Even in wealthier urban areas, the inherent delays to initiate treatment make them clinically ineffective. The technical barriers to safely enable lung clearance of CO are low, making it feasible to provide for widespread availability of the means for early and rapid CO elimination. In any case, early pulmonary CO clearance does not delay or preclude any other treatment, including subsequent treatment with hyperbaric O_2_.

## Supporting Information

CO Model.zip.

## References

[b1] Annane D, Chadda K, Gajdos P, Jars-Guincestre MC, Chevret S, Raphael JC (2010). Hyperbaric oxygen therapy for acute domestic carbon monoxide poisoning: two randomized controlled trials. Intensive Care Med.

[b2] Ayas N, Bergstrom LR, Schwab TR, Narr BJ (1998). Unrecognized severe postoperative hypercapnia: a case of apneic oxygenation. Mayo Clin Proc.

[b3] Baddeley H, Brodrick PM, Taylor NJ, Abdelatti MO, Jordan LC, Vasudevan AS, Phillips H, Saunders MI, Hoskin PJ (2000). Gas exchange parameters in radiotherapy patients during breathing of 2%, 3.5% and 5% carbogen gas mixtures. Br J Radiol.

[b4] Becker HF, Polo O, McNamara SG, Berthon-Jones M, Sullivan CE (1996). Effect of different levels of hyperoxia on breathing in healthy subjects. J Appl Physiol.

[b5] Brown SD, Piantadosi CA (1990). In vivo binding of carbon monoxide to cytochrome c oxidase in rat brain. J Appl Physiol.

[b6] Brown SD, Piantadosi CA (1992). Recovery of energy metabolism in rat brain after carbon monoxide hypoxia. J Clin Invest.

[b7] Bruce EN, Bruce MC, Erupaka K (2008). Prediction of the rate of uptake of carbon monoxide from blood by extravascular tissues. Respir Physiol Neurobiol.

[b8] Buckley NA, Isbister GK, Stokes B, Juurlink DN (2005). Hyperbaric oxygen for carbon monoxide poisoning: a systematic review and critical analysis of the evidence. Toxicol Rev.

[b9] Case RB, Greenberg H, Moskowitz R (1975). Alterations in coronary sinus pO_2_ and O_2_ saturation resulting from pCO_2_ changes. Cardiovasc Res.

[b10] Coburn RF (1970). The carbon monoxide body stores. Ann NY Acad Sci.

[b11] Cronje FJ, Carraway MS, Freiberger JJ, Suliman HB, Piantadosi CA (2004). Carbon monoxide actuates O_2_-limited heme degradation in the rat brain. Free Radic Biol Med.

[b12] Dolan MC (1985). Carbon monoxide poisoning. CMAJ.

[b13] Donald KW, Paton WDM (1955). Gases administered in artificial respiration; with particular reference to the use of carbon dioxide. Brit Med J.

[b14] Douglas TA, Lawson DD, Ledingham IM, Norman JN, Sharp GR, Smith G (1961). Carbogen in experimental carbonmonoxide poisoning. Br Med J.

[b15] Fisher JA, Sommer LZ, Rucker J, Vesely A, Lavine A, Greenwald Y, Volgyesi G, Fedorko L, Iscoe S (1999). Isocapnic Hyperpnea accelerates carbon monoxide elimination. Am J Respir Crit Care Med.

[b16] Goldstein B, Shannon DC, Todres ID (1990). Supercarbia in children: clinical course and outcome. Crit Care Med.

[b17] Gorman DF, Clayton D, Gilligan JE, Webb RK (1992). A longitudinal study of 100 consecutive admissions for carbon monoxide poisoning to the Royal Adelaide Hospital. Anaesth Intensive Care.

[b18] Haldane JS (1895). The action of carbonic oxide on man. J Physiol.

[b19] Henderson Y (1924). Resuscitation: from carbon monoxid asphyxia, from ether or alcohol intoxication, and from respiratory failure due to other causes; with some remarks also on the use of oxygen in pneumonia, and inhalational therapy in general. JAMA.

[b20] Henderson Y, Haggard HW (1920). The elimination of carbon monoxid from the blood after a dangerous degree of asphyxiation, and a therapy for accelerating the elimination. J Pharmacol Exper Ther.

[b21] Henderson Y, Haggard HW (1922). The treatment of carbon monoxide asphyxia by means of oxygen and CO_2_ inhalation. JAMA.

[b22] Henderson Y, Haggard HW, Coburn RF (1921). The acapnia theory, now. JAMA.

[b23] Hill EP, Hill JR, Power GG, Longo LD (1977). Carbon monoxide exchanges between the human fetus and mother: a mathematical model. Am J Physiol Heart Circ Physiol.

[b24] Hunter FT, Mudd SG (1924). Carbon dioxide treatment in acute alcoholic intoxication. Boston Medical & Surgical Journal.

[b25] Juurlink DN, Buckley NA, Stanbrook MB, Isbister GK, Bennett M, McGuigan MA (2005). Hyperbaric oxygen for carbon monoxide poisoning. Cochrane Database Syst Rev.

[b26] Katznelson R, Minkovich L, Friedman Z, Fedorko L, Beattie WS, Fisher JA (2008). Accelerated recovery from sevoflurane anesthesia with isocapnic hyperpnoea. Anesth Analg.

[b27] Katznelson R, Van Rensburg A, Friedman Z, Wasowicz M, Djaiani GN, Fedorko L, Minkovich L, Fisher JA (2010). Isocapnic hyperpnoea shortens postanesthetic care unit stay after isoflurane anesthesia. Anesth Analg.

[b28] Kety SS, Schmidt CF (1948). The effects of altered arterial tensions of carbon dioxide and oxygen on cerebral blood flow and cerebral oxygen consumption of normal young men. J Clin Invest.

[b29] Killick EM, Marchant JV (1959). Resuscitation of dogs from severe acute carbon monoxide poisoning. J Physiol.

[b30] Lemburg P, Sprock I, Bretschneider A, Storm W, Gobel U (1979). A new concept of therapy in accidental intoxications with halogenated hydrocarbons. Vet Hum Toxicol.

[b31] Longo LD, Hill EP (1977). Carbon monoxide uptake and elimination in fetal and maternal sheep. Am J Physiol Heart Circ Physiol.

[b32] McMaster University Division of Emergency Medicine (2006). Should hyperbaric oxygen be used for CO poisoning?. Can J Emerg Med.

[b33] Norman JN, Ledingham IM (1967). Carbon monoxide poisoning: investigations and treatment. Prog Brain Res.

[b34] Norman JN, MacIntyre J, Shearer JR, Smith G (1970). Use of a one-man, mobile pressure chamber in the treatment of carbon monoxide poisoning. Br Med J.

[b35] Pace N, Strajman E, Walker EL (1950). Acceleration of carbon monoxide elimination in man by high pressure oxygen. Science.

[b36] Piantadosi CA (2002). Carbon monoxide poisoning. N Engl J Med.

[b37] Piantadosi CA, Zhang J, Levin ED, Folz RJ, Schmechel DE (1997). Apoptosis and delayed neuronal damage after carbon monoxide poisoning in the rat. Exp Neurol.

[b38] Potkin RT, Swenson ER (1992). Resuscitation from severe acute hypercapnia. Determinants of tolerance and survival. Chest.

[b39] Prisman E, Slessarev M, Azami T, Nayot D, Milosevic M, Fisher J (2007). Modified oxygen mask to induce target levels of hyperoxia and hypercarbia during radiotherapy: a more effective alternative to carbogen. Int J Radiat Biol.

[b40] Rucker J, Fisher JA, Albert RK, Slutsky AS, Ranieri M, Takala J, Torres A (2006). Carbon monoxide poisoning. Clinical Critical Care Medicine.

[b41] Rucker J, Tesler J, Fedorko L, Takeuchi A, Mascia L, Vesely A, Kobrossi S, Slutsky AS, Volgyesi G, Iscoe S, Fisher JA (2002). Normocapnia improves cerebral oxygen delivery during conventional oxygen therapy in carbon monoxide-exposed research subjects. Ann Emerg Med.

[b42] Sasano H, Vesely AE, Iscoe S, Tesler JC, Fisher JA (2001). A simple apparatus for accelerating recovery from inhaled volatile anesthetics. Anesth Analg.

[b43] Scheinkestel CD, Bailey M, Myles PS, Jones K, Cooper DJ, Millar IL, Tuxen DV (1999). Hyperbaric or normobaric oxygen for acute carbon monoxide poisoning: a randomised controlled clinical trial. Med J Aust.

[b44] Sharp GR, Ledingham IM, Norman JN (1962). The application of oxygen at 2 atmospheres pressure in the treatment of acute anoxia. Anaesthesia.

[b45] Smith G (1962). The treatment of carbon monoxide poisoning with oxygen at two atmospheres absolute. Ann Occup Hyg.

[b47] Smith G, Sharp GR (1960). Treatment of carbon monoxide poisoning with oxygen under pressure. Lancet.

[b48] Soley MH, Jump KB, Shock NW (1941). Carbon dioxid therapy. Cal West Med.

[b49] Sommer LZ, Iscoe S, Robicsek A, Kruger J, Silverman J, Rucker J, Dickstein J, Volgyesi GA, Fisher JA (1998). A simple breathing circuit minimizing changes in alveolar ventilation during hyperpnoea. Eur Respir J.

[b50] Somogyi RB, Vesely AE, Preiss D, Prisman E, Volgyesi G, Azami T, Iscoe S, Fisher JA, Sasano H (2005). Precise control of end-tidal carbon dioxide levels using sequential rebreathing circuits. Anaesth Intensive Care.

[b51] Stoller KP (2007). Hyperbaric oxygen and carbon monoxide poisoning: a critical review. Neurol Res.

[b52] Takeuchi A, Vesely A, Rucker J, Sommer LZ, Tesler J, Lavine E, Slutsky AS, Maleck WH, Volgyesi G, Fedorko L, Iscoe S, Fisher JA (2000). A simple “new” method to accelerate clearance of carbon monoxide. Am J Respir Crit Care Med.

[b53] Tesler J (2000). Rates of elimination of carbon monoxide in males and females. MSc Thesis.

[b54] Thom SR (1990). Carbon monoxide-mediated brain lipid peroxidation in the rat. J Appl Physiol.

[b55] Vesely A, Fisher JA, Sasano N, Preiss D, Somogyi R, El Beheiry H, Prabhu A, Sasano H (2003). Isocapnic hyperpnoea accelerates recovery from isoflurane anaesthesia. Br J Anaesth.

[b56] Walton DC, Eldridge WA, Allen MS, Witherspoon MG (1925). Carbon monoxid poisoning: a comparison of the present methods of treatment. Arch Intern Med.

[b57] Weaver LK, Hopkins RO, Chan KJ, Churchill S, Elliott CG, Clemmer TP, Orme JF, Thomas FO, Morris AH (2002). Hyperbaric oxygen for acute carbon monoxide poisoning. N Engl J Med.

[b58] Weaver LK, Howe S, Hopkins R, Chan KJ (2000). Carboxyhemoglobin half-life in carbon monoxide-poisoned patients treated with 100% oxygen at atmospheric pressure. Chest.

[b59] Wolf SJ, Lavonas EJ, Sloan EP, Jagoda AS (2008). Clinical policy: critical issues in the management of adult patients presenting to the emergency department with acute carbon monoxide poisoning. Ann Emerg Med.

