# Supplementary material for: Rapid elimination of CO through the lungs: coming full circle 100 years on
Source: Exp Physiol. 2011 Oct 3;96(12):1262–9. doi: 10.1113/expphysiol.2011.059428 (PMC3274699; doi:10.1113/expphysiol.2011.059428)
Supplement: Supplementary file 2 [file eph0096-1262-SD2.doc]

**Dynamic Carbon Monoxide Simulator for Mother and Fetus**

The simulation model consists of 3 compartments; alveolar gas exchange, and maternal and fetal blood. Tissue carbon monoxide binding with myoglobin is ignored. As a result the model provides a view of the dynamics of CO poisoning, but only an estimate of the levels involved.

**Numerical Controls**

*Model Parameters*

Maternal Oxygen consumption VO2m (ml/min) = 271 ml/min (Hi*ll et a*l., 1977)

Maternal CO production VCOm (ml/min) = 0.015 ml/min (Hi*ll et a*l., 1977)

Maternal FRC (ml)

Maternal blood volume Vb (ml)

Maternal cardiac output Q (ml/min)

Maternal blood CO affinity relative to oxygen Mb

Maternal blood to/from lungs O2 diffusion coefficient, DO2m ml/min/mmHg = 60 ml/min/mmHg (Hi*ll et a*l., 1977)

Maternal blood to/from lungs CO diffusion coefficient, DCOm ml/min/mmHg = 30 ml/min/mmHg

Note that this halves to 15 in hyperoxia compared to 30 in normoxia (Bruce & Bruce, 2003)

Maternal blood CO affinity relative to O2 Mb = 223 (Hi*ll et a*l., 1977)

Fetal Oxygen consumption VO2f (ml/min) = 22 ml/min

Fetal CO production VCOf (ml/min) 0.0006 ml/min (Hi*ll et a*l., 1977)

Fetal blood volume Vf (ml)

Fetal cardiac output Qf (ml/min)

Fetal blood CO affinity relative to oxygen Mf

Fetal blood to/from maternal blood O2 diffusion coefficient, DO2f ml/min/mmHg = 2.7 ml/min/mmHg (Hi*ll et a*l., 1977) but changed to 0.3 to give a lower fetal PO2, which should be 20 mmHg (Hi*ll et a*l., 1977)

Fetal blood to/from maternal blood CO diffusion coefficient DCOf ml/min/mmHg = 1.5 ml/min/mmHg (Hi*ll et a*l., 1977)

Fetal blood CO affinity relative to O2 Mf = 181 (Hi*ll et a*l., 1977)

Time constants:

Alveolar O2 changes O2aTau = 5 min

Alveolar CO changes COaTau = 5 min

Maternal blood O2 changes O2mTau = 10 min

Maternal blood CO changes COmTau = 5500 min

Fetal blood O2 changes O2fTau = 10 min

Fetal blood CO changes COfTau = 100 min

*Model Operation*

Inspired partial pressure of CO PICO (mmHg)

Inspired partial pressure of O2 PIO2 (mmHg)

Ventilation VA (ml/min)

Barometric Pressure PB (mmHg)

*Graphing*

Plot interval (min)

Stop time (min)

**Operation Controls**

**Initialise** erases all data and sets time to zero

**Go** (starts the simulation, which continues to the stop time)

**Stop** (stops the program)

**SAVE ALL** (saves all the data shown in the upper graph to a text file)

The upper graph shows the complete time course of the CO poisoning.

The lower graph allows a comparison of selected traces from the top graph

Once the upper graph display has completed

Select a period of interest starting at the green cursor and ending at the red

Cursor times can be set by dragging the cursors (cursor times shows the values)

Select the variable to be compared

Name this plot

Press **Select** to transfer the data to the lower graph

Up to 5 plots may be displayed for comparison on the mower graph

**Save Selected** (saves all the data shown in the lower graph to a text file)

**Some usage tips**

Right click on any numerical control and from the pop up menus select Data Operations and Reinitialize to Default value to return to the default value.

Scales are set to autoscale but right clicking on any scale and deselecting autoscale can turn off this feature; then maximum and minimum scale values can be entered.

If the cursors are not in view left click on the black (middle) box beside the cursor times and select Bring to Centre or type in a cursor time.

**Exercise**

Start the simulation

Set 0.3 mmHg inspired CO, PICO

Keep all the default values of the parameters

Set ventilation at 6000 ml/min

Set the stop time to 180 minutes (3 hours)

Press Initialise

Press GO

The upper graph displays the time course of the CO poisoning

Now set 0 PICO

Set the stop time to 600 minutes (10 hours)

Press GO

The upper graph displays the washout of CO from the blood

Drag the start (green) cursor to a start time of 3 hours

Drag the end (red) cursor to a stop time of 10 hours

Enter fetal normoxia as a plot name

Choose variable Fetal

Press Select

The normal fetal CO washout from 3 to 10 hours is displayed in the first plot of the lower graph

Press initialise to discard the data of the top graph and set time to zero.

Set 0.3 PICO

Set the stop time to 180 minutes

Press GO

The upper graph displays the time course of the CO poisoning again

Set 0 PICO

Set PIO2 to 650 mmHg

Set the stop time to 600 minutes

Press GO

The upper graph displays the washout of CO from the blood with hyperoxia

Drag the start (green) cursor to a start time of 3 hours

Drag the end (red) cursor to a stop time of 10 hours

Enter fetal hyperoxia as a plot name

Choose variable Fetal

Press Select

The hyperoxic CO washout from 3 to 10 hours is displayed in the second plot of the lower graph

The plots indicate that hyperoxia has markedly decreased the washout time.

**The Model Equations**

The model is simplified as much as possible by making numerous assumptions.

Differential equations are solved by Euler integration using a small dt

(old = previous iteration value, new = next iteration calculated value)

The main assumption is that any current difference of gas transfer between compartments and from gas consumption or production must eventually reach zero. The change is exponential, with a time constant, Tau.

*Variables:*

Time t min

Integration interval dt 0.01 min

Maternal O2Hb % O2Hbm %

Fetal O2Hb % O2Hbf %

Maternal COHb % COHbm %

Fetal COHB % COHbf %

Alveolar partial pressure of O2 PAO2

Maternal partial pressure of O2 PaO2

Fetal blood partial pressure of O2 PfO2

Alveolar partial pressure of CO PACO

Maternal blood partial pressure of CO PaCO

Fetal blood partial pressure of CO PfCO

**The exponential changes are calculated as follows:**

*Alveolar compartment*

Oxygen

Exchange with air VAO2 = VA*(PIO2 - oldPAO2)/(PB-47);

Exchange with blood QAO2 = DO2m*(oldPAO2 - PaO2)/(PB-47);

VAO2 = QAO2 in steady state

This difference declines exponentially with a time constant O2aTau

So each time increment the change is dt*(VAO2 – QAO2)/O2aTau

newPAO2 = oldPAO2 + dt*(VAO2 – QAO2)/O2aTau;

CO

Exchange with air VACO = VA*(PICO - oldPACO)/(PB-47);

Exchange with blood QACO = DO2m*(oldPACO - PaCO)/(PB-47);

VACO = QACO in the steady state

This difference declines exponentially with a time constant COaTau

So each time increment the change is dt*(VACO – QACO)/COaTau

newPACO = oldPACO + dt*(VACO – QACO)/COaTau;

*Maternal blood compartment*

Oxygen

Exchange with alveolar QaO2 = DO2m*(PAO2 - oldPaO2);

Exchange with all tissues VO2m

VO2 = QaO2 in the steady state

This difference declines exponentially with a time constant O2mTau

So each time increment the change is dt*(QaO2 - VO2m)/O2mTau;

newPaO2 = oldPaO2 + dt*(QaO2 - VO2m)/O2mTau;

CO

Exchange with alveolar QaCO = DCOm*(PACO - oldPaCO);

Exchange with all tissues VCOm + VCOf

VCOm + VCOf = QaCO in the steady state

This difference declines exponentially with a time constant COmTau

So each time increment the change is dt*(QaCO + VCOm + VCOf)/COmTau;

newPaCO = oldPaCO + dt*(QaCO + VCOm + VCOf)/COmTau;

*Fetal blood compartment*

Oxygen

Exchange with maternal blood QfO2 = DO2f*(PaO2-PfO2)

Exchange with all tissues VO2f

VO2f = QfO2 in the steady state

This difference declines exponentially with a time constant O2fTau

So each time increment the change is dt*(VO2f – DO2f*(PaO2-PfO2))/O2fTau

newPfO2 = oldPfO2 + dt*(VO2f – DO2f*(PaO2-oldPfO2))/O2fTau;

CO

Exchange with maternal blood QfCO = DCOf*(PaCO-PfCO)

Exchange with all tissues = VCOf

VCOf = QfCO in the steady state

So each time increment the change is dt*(VCOf – DCOf*(PaCO-PfCO))/COfTau

newPfCO = oldPfCO + dt*(VCOf – DCOf*(PaCO-oldPfCO))/COfTau;

**For both blood compartments the Haldane equation must be satisfied:**

(M*PCO/COHb) = (PO2/O2Hb);

Assume: %[O2Hb] is equal 100 – %[COHb] (Cobu*rn et a*l., 1965)

(M*PCO/COHb) = (PO2/(100-COHb))

M*PCO*(100-COHb) = PO2*COHb

100*M*PCO – M*PCO*COHb = PO2*COHb

COHb*(PO2 + M*PCO) = 100*M*PCO

COHb = 100*M*PCO/(PO2 + M*PCO);

*Maternal blood*

COHbm = 100*Mb*PaCO/(PaO2 + Mb*PaCO); %

*Fetal blood*

COHbf = 100*Mf*PfCO/(PfO2 + Mf*PfCO); %

References:

Bruce, E. N. & Bruce, M. C. (2003). A multicompartment model of carboxyhemoglobin and carboxymyoglobin responses to inhalation of carbon monoxide. *J Appl Physiol* **95,** 1235-1247.

Coburn, R. F., Forster, R. E. & Kane, P. B. (1965). Considerations of the physiological variables that determine the blood carboxyhemoglobin concentration in man. *J Clin Invest* **44,** 1899-1910.

Hill, E. P., Hill, J. R., Power, G. G. & Longo, L. D. (1977). Carbon monoxide exchanges between the human fetus and mother: a mathematical model. *Am J Physiol* **232,** H311-323.
